# Antioxidant Properties and Industrial Uses of Edible Polyporales

**DOI:** 10.3390/jof7030196

**Published:** 2021-03-09

**Authors:** Karen P. Quintero-Cabello, Marco A. Lugo-Flores, Patricia Rivera-Palafox, Brenda A. Silva-Espinoza, Gustavo A. González-Aguilar, Martín Esqueda, Rigoberto Gaitán-Hernández, J. Fernando Ayala-Zavala

**Affiliations:** 1 Centro de Investigacion en Alimentacion y Desarrollo, A.C., Carretera Gustavo Enrique Astiazaran Rosas, No. 46, Col. La Victoria, Hermosillo, Sonora CP. 83304, Mexico; karen.quintero.mc18@estudiantes.ciad.mx (K.P.Q.-C.); marco.lugo.mc18@estudiantes.ciad.mx (M.A.L.-F.); patricia.palafox.mc18@estudiantes.ciad.mx (P.R.-P.); bsilva@ciad.mx (B.A.S.-E.); gustavo@ciad.mx (G.A.G.-A.); esqueda@ciad.mx (M.E.); 2 Instituto de Ecologia, A.C., Carretera Antigua a Coatepec, No. 351, El Haya, Xalapa, Veracruz CP. 91073, Mexico; rigoberto.gaitan@inecol.mx

**Keywords:** mushroom, phenolic compounds, vitamins, sociological impact

## Abstract

The content of antioxidant compounds varies within fungal species, and the Polyporales order has been recognized for this property. Numerous antioxidant compounds have been identified in Polyporales fungi, including phenolic compounds, β-glucans, ergosterol, ergothioneine, vitamin C, and tocopherols. Each compound contributes differently to the antioxidant potential of fungi. Besides the health benefits for rural communities caused by fungi consumption, their antioxidant composition attracts the food, cosmetic, and pharmaceutical industries’ interest. In this context, the present review compiles, analyzes, and discusses the bioactive composition of edible fungi of the Polyporales order and its contribution to total antioxidant capacity.

## 1. Introduction

Mushrooms are used as food and medicine, besides having a positive impact on the environment due to their biodegradable function [1]. The order Polyporales consists of more than 1800 species of fungi in the Basidiomycota division. *Ganoderma lucidum*, *Grifola frondosa*, *Taiwanofungus camphoratus*, *Lignosus rhinocerus*, and *Trametes versicolor* are cultivated and marketed as traditional Asian medicine [2]. Some species, such as *Laccocephalum mylittae*, *Lentinus squarrosulus,* and the genera *Laetiporus* and *Sparassis*, are used as food in Asian and African communities. One of the most attractive and studied properties of the Polyporales order is their antioxidant sources.

Interest in the consumption and cultivation of edible mushrooms is millenary, but it has increased in popularity in recent years due to consumer needs regarding health maintenance. Mushrooms are considered a food with high nutritional value; they are rich in fiber, protein, minerals, and low in fat [3]. In addition, bioactive compounds have been identified as phenolic compounds, ergosterol, β-glucans, ergothioneine, vitamin C, and tocopherols [4,5]. These have different properties, such as antibacterial, antiviral, anticancer, and antioxidant properties [6,7]. Some mushrooms are considered functional foods, and they are added to food products to incorporate their bioactive compounds, which can be extracted from fruiting bodies, mycelia, and spores, to be applied in the food and cosmetic industries [8]. All these benefits highlight the potential industrial uses of mushrooms; however, they still represent a scarcely studied kingdom.

The antioxidant potential of fungi will depend not only on the content of bioactive compounds but also on the molecular structure, number, and functional group position [9]. It is also important to consider the molecule’s hydrophilic and lipophilic nature since they will act in different cellular environments. Therefore, the objective of this review was to analyze and discuss the content of bioactive compounds and their contribution to the antioxidant capacity in edible fungi of the order Polyporales.

## 2. Biotechnological Importance of Polyporales

Ethnomycology is a discipline that is dedicated to the study of the relationship between humans and fungi. The beginning of this relationship dates from the year 7000 B.C., with cave paintings in North Africa, specifically in Algeria; engravings of mushroom-like figures were also found in England [10]. In Siberia, Sahara, and Spain, evidence of the use of hallucinogenic mushrooms was found in engraved stones and murals [11]. However, the beginning of ethnomycology as a field of study was stated in 1957 when Gordon Wasson and Valentina Pavlovna published the book Mushrooms, Russia and History, where they described the use of mushrooms in Europe [12]. In several cultures, mushrooms are used in medicine and myths, beliefs, and rituals [13]. This relationship was also found in the Mayan area of Mexico, where hallucinogenic mushrooms were also used in healing-divination rituals, and they were represented in stone figures and clay utensils [10]. Therefore, the beginning of mushrooms’ records indicates mainly their uses as hallucinogenic agents.

Mushrooms in many parts of the world constitute an essential food and economic income [1,14]. The beginning of mushroom consumption dates back to centuries before Christ in China [14]. However, there are different affinities among countries for this tradition; for example, China and Italy’s central area have a strong tradition of mushroom harvesting, while this tradition is weak in the United Kingdom [14]. In Latin America, edible mushroom consumption mainly occurs in Mexico, Guatemala, and Honduras [14]. Their traditional uses have occurred with different purposes, and food is one of them.

The nutritional value and economic revenues of mushrooms are increasing their ethnomycological impact [15]. There has been an increase in cultivated mushroom production, although some countries prefer to collect them from the wild [14]. Nowadays, there is an increase in the commercialization of dietary supplements prepared from mushrooms [14,16]. In 2012, cultivated mushroom production exceeded 20 million tons worldwide [17], with Mexico leading the production in Latin America [1]. *Pleurotus ostreatus* is the main species cultivated in South America, while it is *Lentinula* in Asia, and *Agaricus bisporus* in Europe and North America [1]. The use of mushrooms as food spreads worldwide, and more studies are being performed to improve their growth and cultivation, all to maintain and promote their consumption.

## 3. The Search for Novel Antioxidant Sources and Their Health Benefits

**Figure 1:**
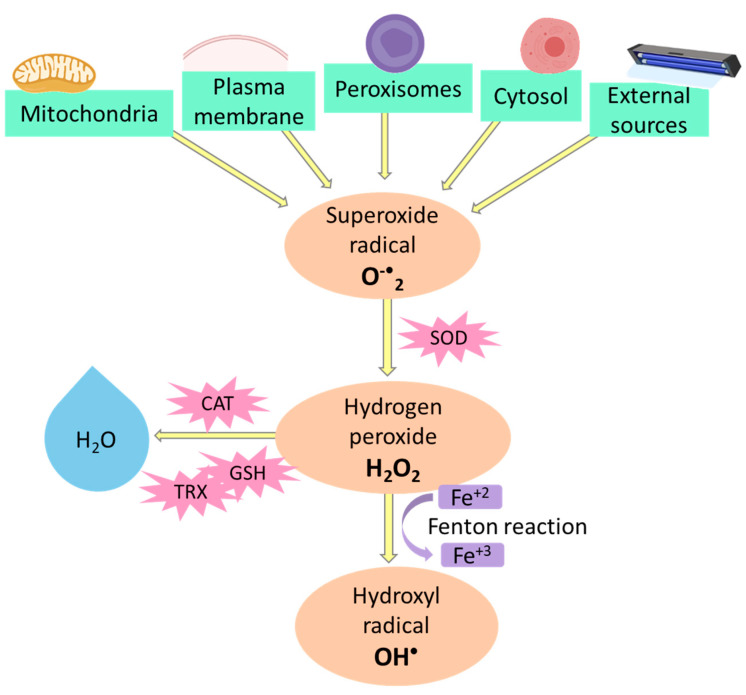
Generation of reactive oxygen species. The superoxide radical is generated by the mitochondria, plasma membrane, peroxisomes, cytosol, and external sources such as U.V. light. Then, it receives an electron to transform into H_2_O_2_; this reaction is catalyzed by the enzyme superoxide dismutase (S.O.D.). H_2_O_2_ can be metabolized by catalases (C.A.T.), glutathione peroxidases (G.S.H.), or thioredoxins (T.R.X.) to convert it to H_2_O. When H_2_O_2_ finds an electron from Fe^+2^ or Cu^+1^, the Fenton reaction produces the hydroxyl radical (O.H.^•^).

A balanced diet includes the consumption of fresh products to reduce the impact of oxidative stress. There are risk factors that increase the possibility of contracting chronic degenerative diseases, such as an inadequate diet and physical inactivity. Additionally, environmental pollution, food additives, tobacco, and pesticides can cause free radicals [18], and these unstable molecules with unpaired electrons need to be stabilized to prevent oxidative stress [9,19]. The body also generates free radicals; for example, they are produced during aerobic respiration in the electron transport chain [18]. Some reactive oxygen species (R.O.S.) include superoxide anion, peroxide anion, peroxyl radicals, and hydroxyl radical [18] (Figure 1).

Oxygen is essential in aerobic metabolism since it participates in various reduction reactions, giving rise to the appearance of R.O.S. and oxidation [20]. The body can neutralize R.O.S. through endogenous antioxidant mechanisms; however, when this capacity is exceeded, oxidative stress is generated, and it causes several diseases [5,18,20,21]. Some exogenous antioxidant agents come in the ingested food [19], and these molecules stabilize free radicals by exchanging one of their electrons, maintaining a balance between oxidants and antioxidant compounds, reducing oxidative stress [5,21]. Consequently, the consumption of foods rich in antioxidants (e.g., mushrooms) contributes to preventing these diseases.

Mushrooms have been used for many years in oriental medicine to prevent and treat various diseases. The antioxidant compounds found in mushrooms include phenolic compounds, amino acids, sterols, polysaccharides, fiber, vitamin C, and tocopherols [5]. Other health benefits provided by these compounds are anti-hypercholesterolemic, antiviral, antibacterial, anti-aging, anti-inflammatory, anticancer agents, and reduction of the toxic effects of chemotherapy and radiotherapy [22]. Their inclusion in the daily diet could also reduce the risk of suffering chronic degenerative diseases [5]. Another way to include mushrooms in our diet is by incorporating them as functional food ingredients to maximize their health benefits [23]. Apart from their food uses, these compounds are also applied in the cosmetic, pharmaceutical, and agricultural industries due to their antioxidant, insecticidal, fungicidal, and bactericidal properties. Therefore, the knowledge of mushrooms’ antioxidant composition is important to propose or explain their functionality in maintaining good health or human benefits.

### 3.1. Vitamin Content: Tocopherols and Vitamin C

**Figure 2:**
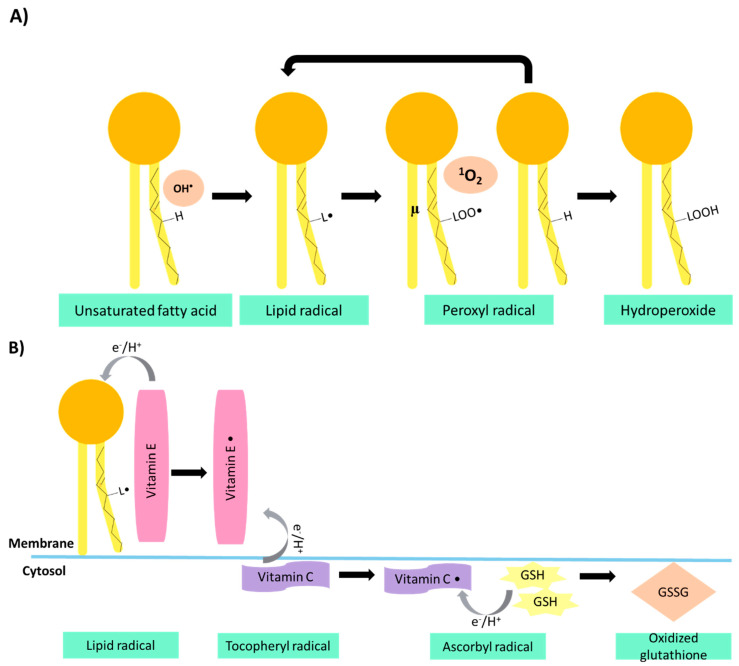
Lipid peroxidation, action, and regeneration of vitamin E. (**A**) Peroxidation begins with the free radical attack against unsaturated fatty acids of the cell membrane, becoming lipid radicals, and in the presence of oxygen, peroxyl radicals are formed. These radicals will attack the nearest fatty acids to form hydroperoxide, generating new lipid radicals, and thus continue the reaction. (**B**) Vitamin E is embedded within the cell membrane, where it donates an electron (e−) and a proton (H +) to the L • and LOO • radicals to neutralize them, becoming α-tocopheryl radicals. This radical is neutralized by the vitamin C, donating an e− and H +, neutralizing the α-tocopheryl radical, generating ascorbyl radicals. Subsequently, two glutathione (G.S.H.) donate an e− and an H + to regenerate vitamin C, becoming oxidized glutathione (G.S.S.G.).

Vitamin E consists of four tocopherols and four tocotrienols, with α-tocopherol being the one with the highest activity [9]. These have a chromanol ring and an isoprene side chain; their differences are in the number of methyl groups, their position in the ring, and the saturated or unsaturated side chain; if the structure has a saturated chain, they are tocopherols, and if it is unsaturated, they are tocotrienols [24,25]. This vitamin is fat-soluble and is embedded within the cell membrane, being the first line of defense against the peroxidation of polyunsaturated fatty acids (Figure 2A) [19,21]. It can donate a hydrogen atom to a free radical, becoming an α-tocopheryl radical, regenerated by vitamin C (Figure 2B) due to their synergistic effect [9]. Four different tocopherols have been reported on edible mushrooms; however, tocotrienols were not identified (Table 1) [26]. In different edible fungi, γ-tocopherol was the most reported tocopherol, in contrast to β-tocopherol, which was identified in fewer species. Regarding the content of total tocopherols, Stojković et al. [4] quantified 0.21 µg/100 g dw (dry weight) in *Meripilus giganteus,* Stojković et al. [27] quantified 104.75 µg/100 g dw in *G. lucidum,* and Omar et al. [28] reported 10 µg/100 g in *Lentinus squarrosulus*. In contrast, Mau et al. [29] did not detect tocopherols in *Coriolus versicolor.*

The synthesis of tocotrienols is from the condensation of homogentisic acid and geranyl-geranyl di-phosphate, catalyzed by the homogentisic enzyme geranyl-geranyl transferase, obtaining 2-methyl-6-geranyl geranyl benzo quinol [25]. On the other hand, the synthesis of tocopherols is from the condensation of homogentisic acid and phytol diphosphate, catalyzed by the enzyme homogentisic phytol transferase, forming 2-methyl-6-phytol-benzoquinol [25]. Afterward, both products undergo methylation and cyclization reactions to form tocotrienols and tocopherols, respectively [25]. The δ-tocopherol is formed by direct cyclization of 2-methyl-6-phytol-benzoquinol, using the enzyme tocopherol cyclase, while its methylation in the C-3 position of the ring produces 2,3-dimethyl 6-phytyl-1,4-benzoquinone, the precursor molecule of γ-tocopherol [33]. Finally, α- and β-tocopherol will be generated by a methylation reaction, catalyzed by tocopherol methyltransferase, which consists of the edition of a methyl group in the chromanol ring of δ- and γ-tocopherol to give α-tocotrienol and α-tocopherol, respectively [33]. Studies have found different factors that increase the synthesis of vitamin E in cultivated mushrooms [34]. An increase in the antioxidant content has been seen in *Pleurotus ostreatus* using sucrose instead of glucose, galactose, or fructose as a carbon source during cultivation [35]. Similarly, a pH of 4.5 and a temperature of 30 °C improved the antioxidant content in this fungus [35].

High vitamin E intake has been associated with a reduced risk of chronic degenerative diseases related to oxidative stress, such as coronary heart disease and cancer [36]. Additionally, its benefits have been reported in Alzheimer’s disease since it reduces the brain’s oxidative stress [37]. This vitamin’s antioxidant properties are extremely attractive for the cosmetic industry; for example, α-tocopherol has proven to be an excellent ingredient in cosmetics as an anti-wrinkle and de-pigmenting agent, capable of inhibiting tyrosinase [8]. Similarly, the antioxidant product developed by Kiyou et al. [38] is made with a mushroom culture medium that contains α-tocopherol and claimed its use in the food and pharmaceutical industries.

On the other hand, vitamin C is a 6-carbon lactone with enediol as a functional group derived from glucose, which is considered essential for humans, so it is necessary to obtain it from the diet [9,39]. It is a water-soluble molecule and highly bioavailable [40]; as explained before, it also contributes to protecting the membranes against lipid peroxidation, eliminating free radicals in the aqueous phase of the cytosol, thus avoiding the initiation of peroxidation [40]. However, the content of vitamin C reported in mushrooms is low or null; for example, Gąsecka et al. [41] reported 0.03 mg of vitamin C per g dw in *G. frondosa*, while Mau et al. [29] did not detect it. On the other hand, *P. ostreatus* showed values of 0.36 mg/g dw [42]. The presence of D-erythroascorbate, an analog of ascorbate and which has similar physicochemical properties, has been reported [43]. This vitamin is produced mainly by plants and some mammals; however, it is not common in mushrooms [43].

The synthesis of vitamin C in plants has been very well studied and begins from D-mannose/L-galactose. In fungi, the synthesis of D-erythroascorbate consists of three steps. It begins with D-arabinose’s oxidation to D-arabinono-1,4-lactone, catalyzed by the enzyme D-arabinose dehydrogenase [44]. Then, it is oxidized to D-erythroascorbate by the enzyme D-arabinono-1,4-lactone oxidase [44]. Its content in cultivated mushrooms can be modified since it has been determined that in cultivation conditions with diffuse light and at low temperatures its content increases, while in the absence of light, it decreases [39,45]. In yeast, vitamin C can be induced by supplying substrates such as L-Galactose and l-1,4-lactones converted into vitamin C [46].

The protective effects of vitamin C against neurodegenerative diseases such as Parkinson’s and Alzheimer’s have been reported [47]; its use has also shown immunomodulatory effects [44]. Therefore, its use can be found in various areas, such as the food, pharmaceutical, and cosmetic industry. Kwak et al. [48] claimed that the mixture of vitamin C and *p*-coumaric acid could be used as an anti-aging ingredient and lightener in cosmetic products. Therefore, the study of vitamin C in mushrooms is of biotechnological interest.

### 3.2. Phenolics Compounds

Phenolic compounds are secondary metabolites formed by aromatic rings and hydroxyl groups, recognized as powerful antioxidants in plants and fungi; these molecules are produced as a defense method against U.V. light, insects, viruses, and bacteria [5,49]. They are a varied group with large structures such as tannins and small structures such as phenolic acids. The latter are divided into hydroxybenzoic acids (example: *p*-hydroxybenzoic and protocatechuic acids) with C6–C1 structure and hydroxycinnamic acids (example: ferulic and caffeic acid) with C6–C3 structures [50]. In food, most phenols are in the form of esters, glycosides, or polymers; for example, hydroxycinnamic acids are bound to proteins, lignin, and cellulose of the cell wall [21]. Consequently, they must be hydrolyzed by intestinal enzymes or colon microbiota to be bioavailable [21]. These compounds’ site of action is at the water–lipid interfaces since their hydrophobicity is between vitamin C and E [49].

The content of total phenols in edible mushrooms has been evaluated, e.g., Puttaraju et al. [51] quantified 4 mg of gallic acid equivalents (G.A.E.)/g dw in *L. squarrulosus* and 3 mg G.A.E./g dw in *Lentinus sajor-caju*. In contrast, Doskocil et al. [2] evaluated the content of total phenols in *Neolentinus lepideus*, where they found 0.082 mg G.A.E./g dw. Different phenolic compounds have been identified and quantified in other species (Table 2), detecting highly variable contents [4]. On the other hand, Cheung et al. [52] reported the correlation between the antioxidant activity and phenolic compounds content in extracts of *L. edodes* and *V. volvacea* [53].

**Figure 3:**
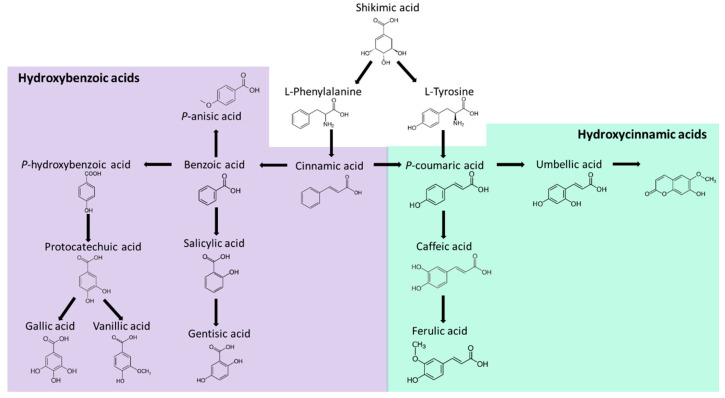
Synthesis of phenolic compounds.

The synthesis of phenolic acids is activated through the shikimate route (Figure 3), where the precursor molecules are the aromatic amino acids phenylalanine and tyrosine. The first step for its synthesis is phenylalanine and tyrosine deamination, producing cinnamic acid and *p*-coumaric [49]. The aromatic rings of cinnamic and *p*-coumaric acid are hydroxylated and methylated to form ferulic and caffeic acid and produce other compounds such as scopoletin [49]. Additionally, the degradation of the side chain of cinnamic acid gives benzoic acid [49]. Then, benzoic acid gives rise to salicylic acid, gentisic acid, *p*-hydroxybenzoic acid, protocatechuic acid, vanillic acid, and others such as *p*-anisic acid [55]. The addition of phenylalanine and tyrosine in cultivated fungi induced an increment of phenolic content [56].

Phenolic compounds have many beneficial effects on health; one of them is their antioxidant activity. Due to this property, it has been reported that they can prevent tumor growth and induce apoptosis; also, they can promote the synthesis of endogenous antioxidants by activating the Nrf2/A.R.E. pathway (NF-E2-related factor 2/Antioxidant responsive element) [21]. These benefits have been claimed by several products made from mushrooms; an example is the functional food developed from *G. lucidum* rich in phenolic compounds [57]. Additionally, phenolic acids from fungi have been used as medications in patients with acute and chronic hypoxic lesions [58].

### 3.3. Ergosterol

Lipids are essential components of the cell membrane, phospholipids predominate, but sterols such as ergosterol are also found [32]. Its presence in the cell membrane allows the stability, permeability, and fluidity of this organelle and has a vital role in response to stress [59,60]. Ergosterol has double bonds at C5 = C6 and C7 = C8 and another double bond at C22 = C23 of its side chain [60]. On the other hand, ergosterol is known as provitamin D since when exposed to U.V. light, it is converted into vitamin D2 through photolysis [61]. The ergosterol content in fungi is heterogeneous among species, despite being the most common sterol; for example, Gąsecka et al. [41] found 0.007 mg/g dw in *S. crispa* and 0.211 mg/g dw in *P. squamosus*. In contrast, Rivera et al. [62] did not detect ergosterol in *G. lucidum*; according to the authors, this may be due to the type of culture medium since this fungus showed high amounts of ergosterol in other studies [32].

The synthesis of ergosterol in edible mushrooms has not been studied enough; it is a complex process that involves numerous enzymes that can be divided into three stages. The first stage starts from 2 Acetyl-CoA molecules that will give rise to mevalonic acid; here, the enzyme H.M.G. reductase (3-hydroxy-3-methyl-glutaryl-CoA reductase) participates, which is the first control point to regulate the route [59]. The second stage consists of mevalonic acid’s phosphorylation until obtaining farnesyl-pyrophosphate, and finally ergosterol is obtained from two molecules of farnesyl-pyrophosphate [59,63]. The ergosterol content in edible mushrooms is usually associated with growth, maturation, hyphal formation, and sporulation characteristics [61]. However, several methods are being applied to increase its content in cultivated mushrooms, such as *A. bisporus*, a highly commercialized species, observing that olive leaf and mushroom stems in the substrate increased the ergosterol content [59].

Ergosterol’s antioxidant capacity reduced cellular carcinogenesis and DNA oxidation by inhibiting free radicals [60]. Patents have also been developed from this compound to use its benefits in food and cosmetics. For example, Oliveira et al. [64] developed cereal/mushrooms flours containing ergosterol, proteins, and vitamins, to formulate functional bakery products. Similarly, Iijima et al. [65] developed a patent for an antioxidant that contains ergosterol peroxide extracted from mushrooms, preventing discoloration of fresh or processed foods beverages. Like the other fungi bioactive compounds, ergosterol can be used in different products in the cosmetic and food industry.

### 3.4. Ergothioneine

Ergothioneine is another antioxidant compound; it is an amino acid produced only by fungi, some cyanobacteria, and mycobacteria [66]. It contains histidine and a sulfur atom in the imidazole ring in its structure [67]; it is a water-soluble compound with antioxidant capacity and acts to protect the mitochondria against oxidative stress [66]. Its content in Polyporales mushrooms is variable; for example, 1.11 mg/g dw was reported in *G. frondosa* and 0.56 mg/g dw in *G. lucidum* [66]. Studies have shown its antioxidant effect in vivo and its protective effect against oxidative damage in cell cultures; also, in humans high amounts have been found in the blood, liver, and kidneys [66,68]. A study conducted by Zhao et al. [69] in *Phylloporia ribis* evaluated the antioxidant capacity of different compounds such as caffeic acid, *p*-hydroxybenzoic, and ergothioneine, among others. This amino acid is the one that obtained the most significant efficacy regarding the inhibition of the DPPH radical. This evidence indicates that ergothioneine can be found in different levels, and therefore its contribution to the antioxidant capacity of mushrooms may vary.

**Figure 4:**
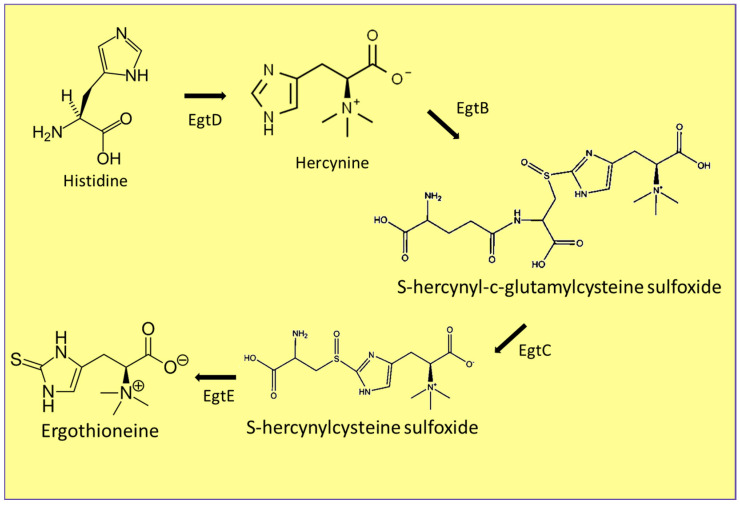
Synthesis of ergothioneine. Ergothioneine biosynthetic proteins EgtB (sulfoxide synthase), EgtC (amidohydrolase), EgtD (methyltransferase), EgtE (hydrolase).

The synthesis of ergothioneine (Figure 4) begins with histidine, which goes through a methylation process, forming hercynine. Then, by the iron-dependent enzyme EgtB sulfoxide synthase, hercynine is transformed into S-hercynyl-c-glutamyl cysteine sulfoxide. Next, it is transformed into S-hercynyl cysteine sulfoxide by the enzyme amidohydrolase EgtC. Finally, ergothioneine is produced by the enzyme EgtE [70,71]. The use of ergothioneine has excellent potential in the industry. In the cosmetic industry, it is used as an anti-aging ingredient [8]. In addition to this, Takashi Abiko [72] developed a patent claiming the formulation of an extract with high amounts of ergothioneine from fungi, with the ability to prevent and treat aging and metabolic syndrome. This patent also claimed the extract’s inhibitory effect on lipases and tyrosinases and antioxidant properties; it also claimed to be an active ingredient in drugs, dermatological products, cosmetics, and food. Hara et al. [73] also created a patent claiming the use of an aqueous extract made from *P. ostreatus* mycelium, rich in ergothioneine and polyphenols, with the ability to suppress discoloration, unpleasant taste, and microbial decay of food.

### 3.5. B-Glucans

The fungi’s cell wall comprises several polysaccharides, mainly glucans; within them are β-glucans forming part of the fiber. The β-glucans are polysaccharides of high molecular weight, consisting of D-glucose monomers linked by β-(1-3) and β-(1-6) bonds, the polysaccharide being the one with the highest bioactivity in mushrooms [74,75]. Several studies have been conducted to determine the presence of β-glucans, such as that by Doskocil et al. [2], who analyzed the content of total β-glucans in extracts of *Lentinus tigrinus* (54.5 mg/g dw) and *Panus conchatus* (21.5 mg/g dw). These polysaccharides are cell wall constituents with bioactive characteristics, including the activation of a non-specific immune stimulation, reduction of blood glucose, cholesterol levels, constipation, and weight control [76].

β-glucans obtained from fungi of the Polyporales order such as *G. frondosa* and *G. lucidum* have been shown to have immunological, anti-inflammatory, and anticancer properties [77]. Patents are also being investigated in different industrial areas are still being investigated; Zhang [78] claimed a method to prepare bread added with *Hericium erinaceus* glucans and with the functionality of reducing blood sugar and lipids. In addition, Li et al. [79] produced nano-membranes with potential uses in medicine to promote wound healing; these membranes were formulated with β-glucans and chitin from *G. lucidum*. Like all the other bioactive compounds mentioned above, β-glucans have a wide range of industry applications, where pharmaceuticals stand out.

## 4. Contribution of Individual Bioactive Compounds to the Total Antioxidant Capacity of Mushrooms

Each compound’s contribution depends not only on its content in the fungus but also on its chemical structure [80]. The antioxidant capacity of a molecule is due to its ability to transfer electrons to unstable molecules, making them less reactive. This characteristic is determined by each molecule, depending on its content and structure. The contribution of bioactive compounds to the antioxidant capacity of various foods such as fruits and natural juices has been evaluated, where it has been found that phenolic compounds are the main contributors [53,80]. Several studies have evaluated the antioxidant potential of different bioactive compounds (Table 3), showing that this capacity is defined by its structure beyond its content in the food matrix.

As described before, phenolic acids can be divided into two groups: hydroxybenzoic acids with a C6–C1 structure and hydroxycinnamic acids with C6–C3 structures [50]. This last group has a higher antioxidant activity due to the presence of the C.H. = CH-COOH group, which facilitates the donation of a hydrogen atom by easily ionizing [50,85]. These compounds’ antioxidant capacity is also due to hydroxyl groups in the *ortho* and *para* position in their structure [9]. Similarly, it is established that the greater the number of hydroxyl groups in the molecule, the greater its antioxidant potential [26,28].

The most-reported phenolic compound in mushrooms is gallic acid; this has a carboxyl group and three hydroxyl groups available to give up hydrogen atoms [9]. These characteristics could explain why it contributes significantly to the antioxidant capacity [80]. Another phenolic acid that is frequently found and contributes significantly to the fungus’ antioxidant capacity is protocatechuic acid; it has a similar structure to gallic acid and has fewer hydroxyl groups [85].

As for polysaccharides, their antioxidant mechanism could be similar to that of phenolic compounds through electron transfer [86]. This capacity can be increased or decreased depending on the combination of monomers and functional groups in their side chain [87]. Additionally, the presence of monosaccharides may favor this property, for example, glucose 1–6 and arabinose 1–4 bonds [87]. Added to the water-soluble compounds is vitamin C, composed of a 5-carbon ring with two chiral centers. Its antioxidant activity is due to its ability to donate electrons through the dissociation of a hydrogen atom and the transfer of one or more electrons to free radicals [9].

On the other hand, in fat-soluble vitamins, we have vitamin E; its structure comprises a 2-methyl-chromanol group attached to a 16-carbon isoprenoid chain, α-tocopherol being the isoform with the highest antioxidant potential. This property is due to the phenolic hydroxyl group since it participates in electrons’ dislocation [9]. Similarly, the methyl groups in the aromatic ring’s ortho position can donate their hydrogen and antioxidant activity [9,88]. Moreover, ergosterol can eradicate radicals in a hydrophobic environment by transferring hydrogen atoms from the C4 position of its structure [60].

Edible mushrooms are food with excellent nutritional value since they are an essential source of proteins, vitamins, minerals, and bioactive compounds. Therefore, their consumption in rural communities brings health benefits, reduces malnutrition problems, and chronic degenerative diseases due to their antioxidant potential.

## 5. Conclusions and Future Trends

Edible species of the Polyporales contain important molecules with antioxidant potential, which could play an essential role in preventing degenerative diseases. The presence of bioactive compounds has a high variability between fungi of different orders and even fungi of the same genus. The standardization of methodologies to analyze fungi composition could contribute to studying species worldwide and achieve better results. Additionally, generating knowledge about species composition can benefit the food, cosmetic, and pharmaceutical industries, developing new products to meet the population’s needs. A great example of this growth is the Asian market, where several mushroom-based patents have been claimed. Finally, this benefit can also be reflected in the economy and the maintenance of traditions in rural communities that use mushrooms. Moreover, more studies are needed to quantify the bioactive compounds’ contribution to edible mushrooms’ total antioxidant capacity in the Polyporales order.

## Figures and Tables

**Table 1:** Composition of tocopherols in Polyporales mushrooms.

Tocopherol	Mushroom Species	Content µg/100g (dw)	Reference
**α-tocopherol**	*M. giganteus*	3	[4]
	*P. squamosus*	4	[30]
	*L. sulphureus*	109.25	[31]
	*G. lucidum*	15.02	[27]
	*G. frondosa*	50	[32]
**β-tocopherol**	*M. giganteus*	9	[4]
	*P. squamosus*	1960	[30]
**δ-tocopherol**	*M. giganteus*	123	[4]
	*L. sulphureus*	18.42	[31]
	*G. lucidum*	89.73	[27]
	*G. frondosa*	40	[32]
**γ-tocopherol**	*M. giganteus*	77	[4]
	*P. squamosus*	N.D.	[30]
	*L. sulphureus*	62.07	[31]
	*G. frondosa*	50	[32]

N.D.—not detected. Dw. Dry weight.

**Table 2:** Phenolic compounds content in Polyporales mushrooms (mg/100g dw).

Mushroom	Identified Phenolic Compound												
	1	2	3	4	5	6	7	8	9	10	11	12	Reference
*G. lucidum*	301.42	522.14	72.55	3.74	1596.01	233.68	138.64	59.16	148.96	-	-	-	[5]
	1.9	N.D.	1.4	N.D.	N.D.	N.D.	N.D.	N.D.	N.D.	0.8	1.8	2.6	[54]
*M. giganteus*	-	1.01	-	-	-	-	2.42	-	0.34	-	-	-	[4]
*S. crispa*	96	34	N.D.	N.D.	N.D.	5	37	N.D.	N.D.	19	66	24	[54]
	0.08	-	-	-	-	-	-	-	-	1.25	-	-	[51]
	N.D.	-	N.D.	-	-	N.D.	N.D.	N.D.	-	0.35	-	N.D.	[41]
*L. sajor caju*	0.03	-	-	-	0.04	0.04	-	-	N.D.	1.51	-	-	[51]
*L. squarrulosus*	0.4	-	-	-	-	-	-	-	0.08	1.92	-	-	[51]
*P. squamosus*	0.035	-	N.D.	-	-	0.012	0.019	N.D.	-	0.052	-	N.D.	[41]
*G. frondosa*	N.D.	-	0.018	-	-	N.D.	N.D.	N.D.	-	N.D.	-	N.D.	[41]

1—protocatechuic acid, 2—*p*-hydroxybenzoic acid, 3—catechin, 4—chlorogenic acid, 5—vanillic acid, 6—syringic acid, 7—*p*-coumaric acid, 8—rutin 9—cinnamic acid, 10—galic acid, 11—pirogalol, 12—quercetin. N.D.—not detected, -—not analyzed.

**Table 3:** Antioxidant capacity of pure compounds.

Compound	TEAC (µmol TE/L)	References
Vitamin C	1000	[81]
α-tocopherol	970	[82]
Ergothionein	870	[83]
Rosmarinic acid	4500	[83]
Gallic acid	3620	[83]
Gentisic acid	0.48	[84]
4-hydroxybenzoic acid	130	[83]
Caffeic acid	1300	[81]
Vanillin	0.335	[84]
Ferulic acid	980	[83]
Salicylic acid	3	[84]

TEAC (Trolox equivalent antioxidant capacity). TE (Trolox equivalent).
